# Impact of Ocean Warming and Ocean Acidification on Larval Development and Calcification in the Sea Urchin *Tripneustes gratilla*

**DOI:** 10.1371/journal.pone.0011372

**Published:** 2010-06-29

**Authors:** Hannah Sheppard Brennand, Natalie Soars, Symon A. Dworjanyn, Andrew R. Davis, Maria Byrne

**Affiliations:** 1 School of Medical Sciences, University of Sydney, Sydney, New South Whales, Australia; 2 National Marine Science Centre, Southern Cross University, Coffs Harbour, New South Whales, Australia; 3 Institute for Conservation Biology, University of Wollongong, Wollongong, New South Whales, Australia; 4 Schools of Medical and Biological Sciences, University of Sydney, Sydney, New South Whales, Australia; Northern Fisheries Centre, Australia

## Abstract

**Background:**

As the oceans simultaneously warm, acidify and increase in *P*_CO2_, prospects for marine biota are of concern. Calcifying species may find it difficult to produce their skeleton because ocean acidification decreases calcium carbonate saturation and accompanying hypercapnia suppresses metabolism. However, this may be buffered by enhanced growth and metabolism due to warming.

**Methodology/Principal Findings:**

We examined the interactive effects of near-future ocean warming and increased acidification/*P*_CO2_ on larval development in the tropical sea urchin *Tripneustes gratilla*. Larvae were reared in multifactorial experiments in flow-through conditions in all combinations of three temperature and three pH/*P*_CO2_ treatments. Experiments were placed in the setting of projected near future conditions for SE Australia, a global change hot spot. Increased acidity/*P*_CO2_ and decreased carbonate mineral saturation significantly reduced larval growth resulting in decreased skeletal length. Increased temperature (+3°C) stimulated growth, producing significantly bigger larvae across all pH/*P*_CO2_ treatments up to a thermal threshold (+6°C). Increased acidity (-0.3-0.5 pH units) and hypercapnia significantly reduced larval calcification. A +3°C warming diminished the negative effects of acidification and hypercapnia on larval growth.

**Conclusions and Significance:**

This study of the effects of ocean warming and CO_2_ driven acidification on development and calcification of marine invertebrate larvae reared in experimental conditions from the outset of development (fertilization) shows the positive and negative effects of these stressors. In simultaneous exposure to stressors the dwarfing effects of acidification were dominant. Reduction in size of sea urchin larvae in a high *P*_CO2_ ocean would likely impair their performance with negative consequent effects for benthic adult populations.

## Introduction

As the oceans warm and absorb increasing amounts of CO_2_, marine biota are faced with a suite of stressors causing major change to marine ecosystems [1]–[2]. Climate change models predict ocean warming by 4°C and a drop in pH by 0.3 to 0.5 units by ca. 2100 [3]–[4]. Ocean acidification is accompanied by a decrease in saturation of the calcium carbonate (CaCO_3_) minerals required to make skeletons and by increased organism *P*_CO2_ (hypercapnia) [5]–[7]. These stressors are likely to have deleterious interactive effects; increased temperature has a stimulatory effect on physiological processes (until thresholds are reached) while hypercapnia has a suppressive, narcotic effect [6]–[7]. In assessing risk to marine biota from climate change it is critical to investigate interactive effects of stressors in multifactorial experiments as this better reflects the real world scenario [1], [7].

Temperature, pH, *P*_CO2_ and CaCO_3_ saturation are among the most important environmental factors controlling the distribution, physiological performance, morphology and behaviour of marine invertebrates [6]–[8]. The projected reduction in CaCO_3_ saturation presents a major challenge to calcifiers in producing their skeletons. Fragile larval skeletons may be the weak link for persistence of some species. For benthic organisms, compromised larval performance has implications for recruitment success and persistence of adult populations [1], [9].

Despite the well known controlling influence of temperature on development and the thermal thresholds exhibited by embryos, investigation of the impacts of climate change on marine life histories has largely focussed on ocean acidification as the sole stressor [10]–[11]. The potential for interactive effects of ocean warming and CO_2_ driven acidification on larval development remains largely unexplored. Single stressor studies of *P*_CO2_ induced acidification show impaired development in echinoderm and mollusc larvae reared in the acidified/elevated *P*_CO2_ conditions projected for 2100 [13]–[17]. In the single study of interactive effects of ocean warming and acidification/*P*_CO2_ on early development, echinoid cleavage stage embryos and gastrulae were most affected by temperature [18]. Decreased pH (adjusted with mineral acid) and increased temperature both exert a negative effect on calcification in oyster larvae [19]. For post-larval and juvenile calcifiers transplanted to laboratory mesocosms or adults resident near CO_2_ vents, acidification and warming both exert negative effects with increased temperature of greatest concern [20]–[22].

We investigated the interactive effect of ocean warming and acidification on the larvae of *Tripneustes gratilla*, a sea urchin widely distribution throughout the Indo-Pacific [23]. This species is ecologically important, especially in sea grass habitats and is a food source with good potential for aquaculture [23]–[27]. The interactive effects of climate change stressors were investigated in *T. gratilla* reared in near future conditions in embryos fertilised in experimental conditions. Fertilization in this species is robust to near-future ocean warming and acidification [28]. We focused on the larval stage because it produces a fragile calcite skeleton, and because this life stage has a planktonic period of days or weeks in the water column where seawater chemistry and temperature have a major impact on development. Echinoplutei produce calcite rods that support their body, and function in swimming and feeding. Arm length, and thereby calcite rod growth, has a direct influence on the efficiency of larval feeding and on vulnerability to predation [29]–[30]. Temperature has a major influence on development in shortening the planktonic period, an effect that decreases predation pressure and also alters connectivity between populations [31]–[32].

Our experiments were placed in a climate and regionally relevant setting for the SE Australia climate change hot spot (warming: +3–6°C; acidification: −0.3–0.5 pH units) [4], [33]. Due to changes in ocean circulation, this region is warming considerably faster than the global average [33]. Fertilisation in *T. gratilla* and other echinoids is robust to climate change stressors [28], [34]–[35] and larval survival in this species decreases at pH 7.0 [15]. We predicted a) that development would be facilitated by warming up to a threshold; b) that skeletogenesis would be impaired by increased acidification/*P*_CO2_ and, c) due to temperature enhancement of metabolic processes, increased temperature would counter the negative effects of decreased calcite and aragonite saturation on skeletogenesis.

## Results

### Normal Development

**Figure 1:**
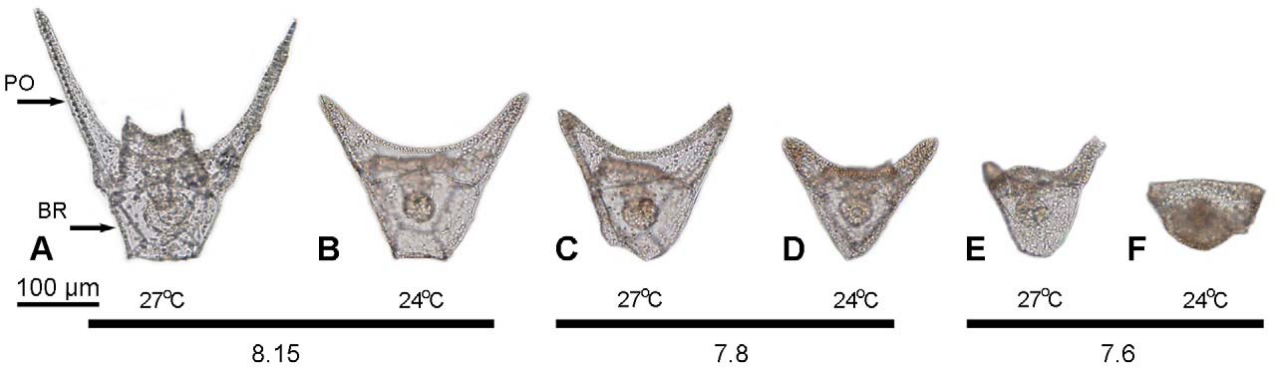
*Tripneustes gratilla* larvae reared for 5 days in 3 pH and 2 temperature treatments. A-B. Control pH 8.15, largest larvae were from +3°C (27°C) treatments. PO, post oral arms; BR, body rod. C-D. pH 7.8. E-F. pH 7.6. With increased acidity/*P*_CO2_ larval size decreased and there was an increase in abnormal development.

**Figure 2:**
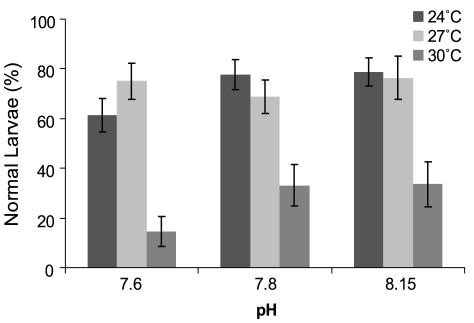
Percentage of normal *T. gratilla* larvae. Percentage of normal *T. gratilla* larvae in nine treatments (3 pH×3 temperature levels) in the larvae from 3 females. See Table 4 for *P*_CO2_, Ωcalcite and Ωaragonite conditions.

The range of morphology of *T. gratilla* seen in the treatments on day 5 is shown in Fig. 1. There was a significant effect of temperature and pH/*P*_CO2_, on the percentage of normal larvae (temp: *p* = <0.003; pH: *p* = 0.04; Table 1, Fig. 2). The upper warming, +6 (30°C) approached the thermal tolerance of development (<30% normal larvae). The percentage of normal larvae was highest (>85%) in the control pH/*P*_CO2_ and 24°C and 27°C treatments. At 24°C and 27°C, the percentage of normal larvae was >60% across all treatments. The slight decrease in normal development at 27°C pH 7.8 (Fig. 2) was not statistically significant (Table 1). Egg source was significant (*p* = <0.0001), but did not interact with the other factors.

**Table 1:** ANOVA of percentage normal *Tripneustes gratilla* larvae reared in temperature (temp) and pH/*P*_CO2_ (as fixed factors) treatments, with egg source (female) as a random factor, and Tukey-Kramer post-hoc tests (TK).

Source	df	MS	*F*	*p*	TK
Temp*	2	3.595	35.3	0.0029	(24, 27) >30
pH*	2	0.230	8.0	0.0402	(8.15, 7.8) (7.8, 7.6), 8.15 >7.6
temp × pH	4	0.126	2.2	0.1598	
Female*	2	1.348	30.6	<0.0001	
Temp × female	4	0.101	2.3	0.0691	
pH × female	4	0.028	0.7	0.6243	
temp × pH × female	8	0.057	1.3	0.2595	
Residual	54	0.044			
Total	80				

*Significant, *p*<0.05; df, degrees of freedom; MS, mean square; *n* = 3 replicates for each of 3 females.

**Figure 3:**
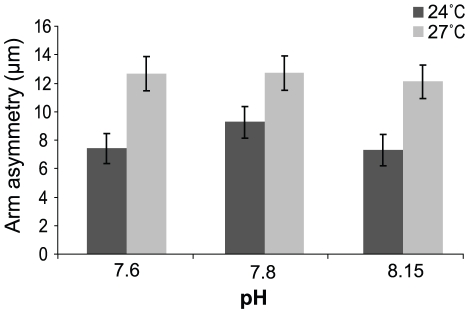
Mean arm asymmetry in *T. gratilla* larvae. Mean arm asymmetry in *T. gratilla* larvae in six treatments (3 pH ×2 temperature levels) in 35 larvae from each of 3 females (*n* = 3, ±SE). See Table 4 for *P*_CO2_, Ωcalcite and Ωaragonite conditions.

The mean difference in PO arm length (larval asymmetry) differed among the 24°C and 27°C treatments (*p* = 0.001; Table 2, Fig. 3). Asymmetry was most marked in the faster growing larvae reared at +3°C (27°C). Arm asymmetry was not significantly affected by pH/*P*_CO2_ (*p* = 0.545; Table 2). There was no interaction between factors.

**Table 2:** ANOVA on difference in PO arm length (asymmetry) data for *Tripneustes gratilla* larvae reared in temperature (temp) and pH/*P*_CO2_ (as fixed factors) treatments.

Source	df	MS	*F*	*p*
pH	2	0.4	0.6	0.545
Temp*	1	0.9	17.1	0.001
pH × temp	2	0.02	0.4	0.687
Residual	12	0.05		
Total	17			

*Significant, *p*<0.05; df, degrees of freedom; MS, mean square; *n* = 3 from the means of 35 larvae per female.

### Larval Growth

**Figure 4:**
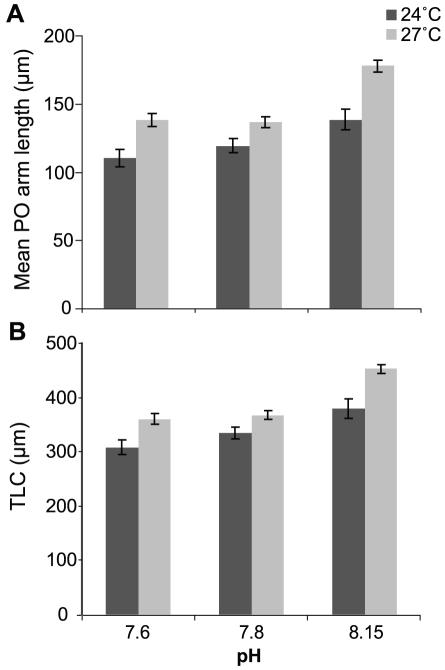
Postoral arm and total calcite rod length in *T. gratilla* larvae. A. Mean post oral (PO) arm length and B. total length of calcite rods (TLC) of *T. gratilla* larvae in six treatments (3 pH ×2 temperature levels) in 35 larvae from each of 3 females (*n* = 3, ±SE). See Table 4 for *P*_CO2_, Ωcalcite and Ωaragonite conditions.

Five-day echinoplutei had well developed PO arms and these were the longest skeletal element (Fig. 1). Arm length significantly increased with temperature and decreased in acidified conditions (temp: *p* = <0.0001; pH: *p* = <0.0001; Table 3, Fig. 1,4). The PO arms were longer in the +3°C (27°C) treatment than in controls (24°C), 178.1 µm (SD = 3.9, *n* = 35) and 138.7 µm (SD = 2.6, *n* = 35), respectively. Larvae reared at pH 7.6 and pH 7.8 had smaller PO arms when compared with those reared at control pH across both temperature treatments (TK 7.6 = 7.8<8.15; Table 3, Fig. 1). However a +3°C warming diminished the negative effects of low pH/high *P*_CO2_. This is seen in the similar PO arm length of larvae reared at 27°C/pH 7.6 and 27°C/pH 7.8 and those reared in control temperature and pH (Fig. 4). As TLC is largely comprised of the PO arms, this measure followed a similar pattern (temp: *p* = 0.0001; pH: *p* = <0.0001; Table 3; Fig. 4). There was no interaction between temperature and pH (Table 3).

**Table 3:** ANOVA of mean post oral arm length (PO) and total length of calcite rod (TLC) data for *Tripneustes gratilla* larvae reared in temperature (temp) and pH/*P*_CO2_ (as fixed factors) treatments, and Tukey-Kramer post-hoc tests (TK).

Parameter	Source	df	MS	*F*	*p*	TK
PO	pH*	2	2105	28	<0.0001	8.15>(7.8, 7.6)
	Temp*	1	3621	48	<0.0001	
	pH × temp	2	183	2.4	0.1297	
	Residual	12	75			
	Total	17				
TLC	pH*	2	11136	27	<0.0001	8.15>(7.8, 7.6)
	Temp*	1	12448	30	0.0001	
	pH × temp	2	624	1.5	0.2623	
	Residual	12	416			
	Total	17				

*Significant, *p*<0.05; df, degrees of freedom; MS, mean square; *n* = 3 from the means of 35 larvae per female.

## Discussion

In this first study of the effects of simultaneous exposure to warming and CO_2_ driven acidification on calcification in marine invertebrate larvae reared in experimental conditions from fertilization, we show the positive and negative effects of these stressors. Larval growth in *T. gratilla* was positively correlated with increased temperature across all pH treatments until the thermal threshold was breached, supporting our first prediction. In contrast, larval growth was negatively correlated with increased acidity/*P*_CO2_ and decreased calcite and aragonite saturation, resulting in smaller larvae, supporting prediction two. Warming countered to some extent the negative effects of acidification, providing some support for prediction three.

Temperature is considered to be the primary environmental factor controlling the physiology, phenology, planktonic larval duration and biogeography of marine invertebrates [31]–[32], [36]–[37]. The response of *T. gratilla* larvae to increased temperature reflected the typical pattern seen in echinoids and other invertebrates with a balance between facilitation at certain levels of warming and failure at upper thermal limits [32], [37]–[41]. Temperature is well known to control the pace of development in marine larvae. A +3°C warming resulted in faster growth and increased size in *T. gratilla* larvae.

Developmental thermotolerance varies greatly between echinoids with a +4°C warming above ambient approaching the thermal limit of many species [34]. Embryogenesis in tropical species such as *T. gratilla* and *Echinometra* spp. is more robust to thermal increase [37], [38], [41]. For *T. gratilla*, 30°C approximates the lethal threshold for development in both high and low latitude populations [41, this study]. This thermotolerance will facilitate persistence of *T. gratilla* and possible poleward spread from its southern limit in Australia where mean SST are not expected to go beyond ∼28°C by 2100, but does not bode well for tropical populations where SSTs will continue to exceed 30°C [4], [33].

A +3°C warming enhanced larval growth of *T. gratilla* across all pH/*P*_CO2_ treatments, to some extent buffering the negative effects of these factors. Larvae reared at low pH had significantly shorter PO arms than those reared in control pH suggesting suppressed calcification, as in a previous study [15]. This reduction in size is likely due to hypercapnic suppression of metabolism causing delayed development and decreased availability of CaCO_3_ for skeletogenesis. Decreased biomineralisation in response to near-future (ca. 2100) acidification is reported for other echinoid larvae [13], [14], [17].

Increased ocean acidity, hypercapnia and decreased carbonate mineral saturation are inextricably linked and are all likely to exert negative effects on larvae. This may be through direct pH effects on metabolic systems such as those involved with calcite precipitation (eg. carbonic anhydrase) and cellular protection (eg. heat shock proteins) and direct hypercapnic suppression of metabolism [6], [7], [17], [42]–[44]. Although calcite and aragonite remained saturated in our treatments (Ωcalcite 1.6–5.9, Ωaragonite 1.1–4.0), they decreased markedly at low pH, with aragonite approaching minimal levels. At Ω <1, seawater becomes corrosive causing dissolution and impaired skeleton deposition in larval and adult sea urchins [8], [44].

Calcification in sea urchin larvae occurs internally, under a different chemical environment than surrounding seawater through an amorphous phase of CaCO_3_ that would dissolve if exposed to low pH seawater [45], [46]. Echinoderms and other invertebrates adjust internal pH through accumulation of bicarbonate ions [42], which would alter internal calcite conditions. Some echinoderms and other benthic calcifiers may be able to maintain an alkaline environment at the internal mineralization site despite reduced external pH [46]. What is poorly understood is how the availability of carbonate ions in the ocean affects this process.

The effects of ocean acidification on marine calcifiers vary among phyla, species, life history stages and latitudes/habitats. With regard to the pelagic life stage, some larvae show deleterious effects of near future *P*_CO2_ driven acidity, while others, even closely related species are more robust [16], [34], [47]. Embryos may be most vulnerable to warming while larvae that survive early mortality bottlenecks may be more affected by acidification [13], [15], [18], [34], [47]. Regional settings for projected change are also a crucial consideration. Cold high latitude waters will become carbonate under-saturated first and so high latitude calcifiers may be most vulnerable to ocean acidification [5]. However, a recent study showed that Antarctic echinoplutei were less affected by acidification than temperate and tropical counterparts [15].

Despite the pervasive effect of ocean warming on development, this factor is rarely considered in studies of climate change impacts. Larval performance may differ in experiments when temperature is brought into the mix of factors assessed. For regions with significant warming such as SE Australia, temperature is the most immediate and contemporary climate change stressor. Many progeny will not reach the calcified larval stage in a warm ocean, regardless of pH/*P*_CO2_ changes [18]. Our data for *T. gratilla* were from embryos fertilised and reared in experimental conditions to the larval stage. The larvae were from the subset of survivors available for measurement. With regard to comparisons between climate change stressor studies, some studies translocate embryos fertilised in present day conditions to experimental treatments and others rear embryos from the outset in experimental conditions [34]. Experimental outcomes may differ between these approaches, the latter being more realistic.

While our results clearly showed the effects of climate change stressors on larval development, egg source also exerted a significant influence. We did not set out to test maternal effects, but it is important to be cognisant of this factor in considering the larval responses. Maternal provisioning influences larval tolerance and ecological outcomes for invertebrate larvae [48]–[49].

With respect to the benthic life phase of marine calcifiers, numerous studies investigate the response of juveniles or adults sourced from field collections or aquaculture translocated from present day to acidified conditions [21]–[22], [44], [46], [50]–[53]. These studies show varied responses, decreased calcification in some species, no change in others and increased calcification in others. A study of arm regeneration in an ophiuroid showed increased calcification at low pH [53]. Simultaneous exposure to warming and acidification resulted in increased growth in juvenile sea stars [52]. The contrasting responses among species are likely to be due to differences in calcifying systems [46] and the environmental history of the organisms prior to being placed in treatments.

We focussed on the pelagic life phase because this is the crucial dispersal stage and is considered to be most vulnerable to environmental perturbations [47]. The thin arm rods of echinoderm plutei are essential for feeding, swimming and protection from predation and feeding success is related to arm length [29]–[30]. Smaller larvae with a longer planktonic duration are more vulnerable to predation in a changing ocean, decreasing chances of survival and recruitment [54]. Projected near future ocean change may result in a major bottleneck for marine life histories with negative flow on effects for the integrity of benthic populations and communities [1], [2], [9]. Calcifying taxa across many phyla play important roles in marine ecosystem function as bioturbators and keystone species and, on a larger scale, biocalcification plays a critical role in the carbon cycle [55]. Negative impacts on calcifiers in a changing ocean have far-reaching implications for biodiversity and ocean health.

## Materials and Methods

### Specimen collection and spawning

*Tripneustes gratilla*, collected near Coffs Harbour, New South Wales (30°12.5'S. 153°16.1'E.), were maintained in flow-through aquaria (∼3500 L) at ambient temperature (∼24°C). They were induced to spawn by injection of 2–3 ml 0.5 M KCl. The eggs of three females were spawned into 500 ml beakers of filtered seawater (FSW 0.2 µm). Sperm were collected dry using pipettes. Before use, the eggs were checked for shape and integrity and sperm were checked for motility. The eggs of each female were fertilised by sperm from multiple males. Each experiment was undertaken with independent sources of gametes with replication based on the three females.

For each egg source ca. 2000 eggs (∼20 eggs ml^−1^) were placed in rearing containers (100 ml), three for each temperature-pH treatment (see below), in flow-through experimental FSW (flow rate ca. 0.13 ml sec^−1^, 300–400 turnovers day^−1^) for 20 minutes prior to the introduction of sperm. The containers had a window cut from each side as an overflow and a 45 µm mesh set back from the overflow to retain eggs. The number of sperm required to achieve a sperm to egg ratio of ca. 1000:1, was determined through haemocytometer counts. The sperm was briefly activated (1–2 sec) in experimental FSW prior to addition to containers holding eggs. The flow-through system was turned off (5 min) during fertilisation and was then turned back on to remove excess sperm. This fertilisation procedure was repeated in separate experiments with the eggs of the three females.

The embryos were reared in experimental conditions to the 5 day echinopluteus stage and were not fed to avoid the potentially confounding influence of algal introduction. *Tripneustes gratilla* embryos have substantial maternal energetic reserves with a long (8 day +) facultative feeding period during which development proceeds in the absence of exogenous food [49]. We chose the 5 day endpoint because by this stage the larvae have well developed arms for measurement and are not nutritionally limited.

### Experimental treatments and rearing

The embryos were reared in experimental flow-through FSW in three temperature (control = 24°C, +3°C, +6°C) and three pH (control = 8.15, −0.3, −0.5 pH units) levels in all combinations with three containers of embryos per treatment. Experimental pH was adjusted using an automatic CO_2_ injection system. Two pH controllers (Tunze), set at pH 7.6 and pH 7.8, were attached to two header tanks (60 L). The controllers, pH probes, solenoid valves and gas cylinders were connected in series and injected pure CO_2_ gas into the header when required, where it was dissolved using a vortex mixing device (Red Sea). The header tanks were continuously bubbled with air to aid mixing and to maintain dissolved oxygen (DO) >90%. A constant volume was maintained in the headers using a float valve. A control header was bubbled with air only. This water was fed into sub-header tanks (20 L) where it was warmed to the required temperature, +3°C (27°C) and +6°C (30°C), using aquarium heaters or unmanipulated for the ambient control. Seawater was delivered to rearing containers using irrigation drip valves. Temperature, pHNBS, DO and salinity at the level of the experimental containers with developing embryos and larvae were measured daily with a WTW multiprobe. Filtered ambient in flow water was not manipulated and had a mean temperature 23.51°C (SE = 0.04, n = 10, Range 23.3–23.8°C) and mean pH 8.13 (SE  = 0.004, n = 10, Range pH 8.08–8.16). The experimental water conditions measured at the level of the rearing containers remained stable (+3°C: Mean 26.13°C, SE = 0.14, Range 25.6–26.6°C; +6°C: Mean 30.35°C, SE = 0.25, Range 29.1–31.9°C) (pH −0.3 units: Mean 7.8, SE  = 0.006, Range pH 7.76–7.87; pH −0.5 units: Mean 7.61, SE  = 0.004, Range pH 7.58–7.65). Total alkalinity (TA 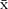 =  2427.1, SE = 5.2, *n* = 4) was determined by potentiometric titration (CSIRO, Hobart). Experimental *P*_CO2_ and calcite and aragonite saturation values (Table 4) were determined from TA, pHNBS and salinity data using CO2SYS [56]. Data for both carbonate minerals were calculated because the saturation state of echinoderm magnesian calcite may be close to that of aragonite [57].

**Table 4:** Temperature (T), pH, *P*_CO2_, and calcium carbonate saturation conditions in the nine experimental treatments.

T	24°C			27°C			30°C		
pH	8.15	7.8	7.6	8.15	7.8	7.6	8.15	7.8	7.6
***P*_CO2_**	448 (3)	1142 (8)	1886 (13)	455 (3)	1169 (8)	1938 (14)	460 (3)	1196 (9)	1990 (14)
**Ωcalcite**	5.1 (0.00)	2.6 (0.02)	1.7 (0.02)	5.5 (0.05)	2.8 (0.03)	1.9 (0.20)	5.9 (0.05)	3.1 (0.03)	2.0 (0.02)
**Ωaragonite**	3.4 (0.03)	1.7 (0.02)	1.1 (0.01)	3.7 (0.03)	1.9 (0.02)	1.2 (0.01)	4.0 (0.04)	2.1 (0.02)	1.4 (0.01)

Mean (SEM) *n* = 4

### Development

Specimens from each rearing container (*n* = 100–200, where available) were placed in 1.5 ml tubes containing 10% formaldehyde-FSW for 10 min, followed by rinse in 70% ETOH in FSW. The first 30 specimens removed randomly from each tube were examined microscopically to score the percentage of normal development. Thus there were three replicate data points per female for this analysis (i.e. 3 containers X 3 temp X 3 pH). Normal larvae were defined as echinoplutei with two arms and a trapezoidal/triangular body (Fig. 1), including larvae with minimal asymmetry (i.e. one arm <30% longer than the other). Abnormal specimens included larvae with marked asymmetry (one arm ≥30% larger than the other), armless arrested larvae and arrested embryos (Fig. 1).

### Larval growth

Larval growth was documented in an image analysis study of photographs of larvae reared at 24°C and 27°C. The 30°C treatments were excluded due to high mortality and insufficient larvae to measure. Haphazardly selected plutei positioned flat to the plane of focus were photographed using a digital camera mounted on a compound microscope. For each female 35 larvae (taken across the three rearing containers) from each treatment were measured using Image J (NIH, USA). Thus a total of 630 larvae were used (3 females ×35 larvae ×3 pH×2 temperatures). For each larva the length of the two post oral (PO) arms body rods (BR) were measured. The mean length of the two PO arms was determined and the difference in their length was calculated as a measure of arm asymmetry. Total length of calcite rods (TLC), determined as the sum of all skeletal elements was used as a proxy for biocalcification.

### Statistics

For the percentage of normal development data where larvae from three separate rearing containers were scored per female a three factor ANOVA with pH and temperature as fixed orthogonal factors and egg source as a random factor was used. Percentage data were arcsine transformed prior to analysis. Homogeneity of variance was checked using Cochran’s test. For the data on difference in PO arm lengths (arm asymmetry), PO length and TLC where a single mean data point derived from 35 larvae sourced from across 3 rearing containers was determined, a two factor ANOVA with pH and temperature as fixed factors was used. The raw data on arm asymmetry was heterogeneous and was ln(*x*) transformed prior to analysis to meet the assumptions of ANOVA. Normality was confirmed by plotting residuals against normal distributions. Where treatments differed, Tukey-Kramer (TK) post-hoc tests were conducted to detect differences amongst means. For the mixed model ANOVA on the percentage normal data we ran the TK test using the interaction term MS and the residual MS and note that the results were identical. All statistics were carried out using NCSS 2007 (V 17).
